# Endoscopic closure of a refractory post-surgical pouch by mucosal resection and suturing

**DOI:** 10.1055/a-2743-9993

**Published:** 2025-12-08

**Authors:** Abdeldjalil Sais, Jérôme Rivory, Florian Rostain, Alexandru Lupu, Olivier Monneuse, Jean Grimaldi, Mathieu Pioche

**Affiliations:** 1 639305 Department of Gastroenterology and Endoscopy, Groupement Hospitalier Portes de Provence (GHPP), Montélimar, France; 2 Department of Gastroenterology and Endoscopy, Hôpital Edouard Herriot, Hospices Civils de Lyon, Lyon, France; 3 Department of Emergency and General Surgery, Hôpital Edouard Herriot, Hospices Civils de Lyon, Lyon, France

Complex surgical reconstructions can lead to challenging anatomical complications 1 . We present the case of a 22-year-old female patient with a history of a suicide attempt by caustic ingestion, resulting in an esogastrectomy and a retrosternal coloplasty with an esophago-ileal anastomosis. Post-operatively, she developed severe dysphagia and regurgitation due to a large diverticular pouch at the anastomosis, creating a sump effect 2 .

Initial endoscopic treatments, including the placement of an AXIOS stent and subsequent balloon dilation up to 20 mm, failed to provide clinical improvement. The persistence of symptoms confirmed that the mechanical issue was the pouch itself, which sequestered food, rather than a simple stricture.

A novel endoscopic procedure was performed to obliterate the pouch ( Video 1 ). The technique involved performing multiple endoscopic mucosal resection (EMR) on the opposing walls of the diverticulum to denude the mucosa ( Fig. 1 **a** ). Following this, the de-epithelialized surfaces were sutured together using an endoscopic suturing device SutuArt Olympus ( Fig. 1 **b** ), effectively closing the pouch and eliminating the residual cavity ( Fig. 1 **c** ).

**Fig. 1:**
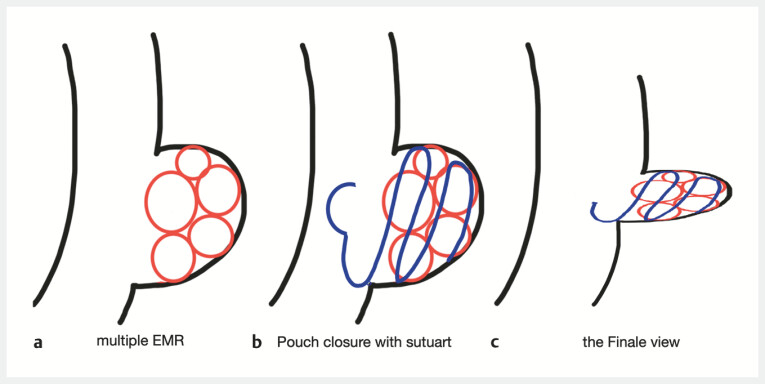
A schematic procedure of an endoscopic pouch closure using a combined mucosal resection and suturing. **a** Multiple EMR. **b** Pouch closure with Sutuart. **c** The final view. EMR, endoscopic mucosal resection.

Endoscopic closure of a large, symptomatic post-surgical pouch using a combination of EMR and endoscopic suturing to achieve complete obliteration. EMR, endoscopic mucosal resection.Video 1

The procedure was successful with no immediate complications ( Fig. 2 **a, b** ), resulting in immediate clinical improvement and allowing for the patientʼs discharge the following day. A 3-month follow-up upper gastrointestinal contrast study subsequently confirmed the complete resolution of the pouch, showing an unobstructed flow of contrast into the distal bowel ( Fig. 2 **c** ). This case demonstrates how a minimally invasive endoscopic approach combining EMR and suturing can successfully manage a complex post-surgical complication, offering a viable alternative to high-risk revisional surgery.

**Fig. 2:**
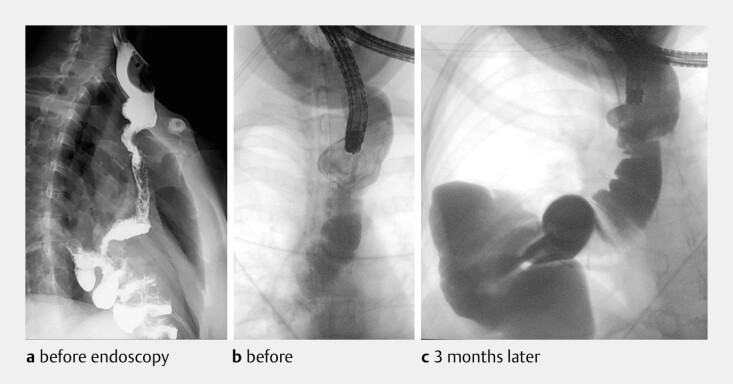
Fluoroscopic sequence showing the post-surgical pouch before and after endoscopic obliteration. **a, b** Pre-procedural imaging demonstrates the significant stasis of the contrast material within the large diverticular pouch. **c** The planned 3-month post-procedural study is intended to confirm the complete resolution of the pouch, with an unobstructed flow of contrast into the distal bowel.

Endoscopy_UCTN_Code_TTT_1AO_2AP

## Notes

Endoscopy E-Videos https://eref.thieme.de/e-videos
*E-Videos* is an open access online section of the journal *Endoscopy* , reporting on interesting cases and new techniques in gastroenterological endoscopy. All papers include a high-quality video and are published with a Creative Commons CC-BY license. Endoscopy E-Videos qualify for HINARI discounts and waivers and eligibility is automatically checked during the submission process. We grant 100% waivers to articles whose corresponding authors are based in Group A countries and 50% waivers to those who are based in Group B countries as classified by Research4Life (see: https://www.research4life.org/access/eligibility/ ). This section has its own submission website at https://mc.manuscriptcentral.com/e-videos .
